# P-513. Outcomes of antenatal antiviral suppressive therapy at 36 weeks gestation in a large cohort of pregnant persons with genital herpes simplex virus

**DOI:** 10.1093/ofid/ofaf695.728

**Published:** 2026-01-11

**Authors:** Tara L Greenhow, Zahra Samiezade-Yazd, Lea Bornstein, Mara Greenberg, Beverly Young, Tran H P Nguyen

**Affiliations:** Kaiser Permanente Northern California, San Francisco, CA; Kaiser Permanente Northern California, San Francisco, CA; Kaiser Permanente Northern California, San Francisco, CA; Kaiser Permanente Northern California, San Francisco, CA; Kaiser Permanente Northern California, San Francisco, CA; Kaiser Permanente Northern California, San Francisco, CA

## Abstract

**Background:**

To decrease rates of cesarean section for active herpes simplex virus (HSV) genital lesions and HSV transmission to infants, several international organizations recommend offering mothers with a history of genital HSV antiviral prophylaxis at or beyond 36 weeks gestation. In a large integrated health care system, we aimed to determine the effect of antenatal antivirals in all pregnant persons with a history of genital herpes and / or genital lesions at delivery.

**Table 1:**
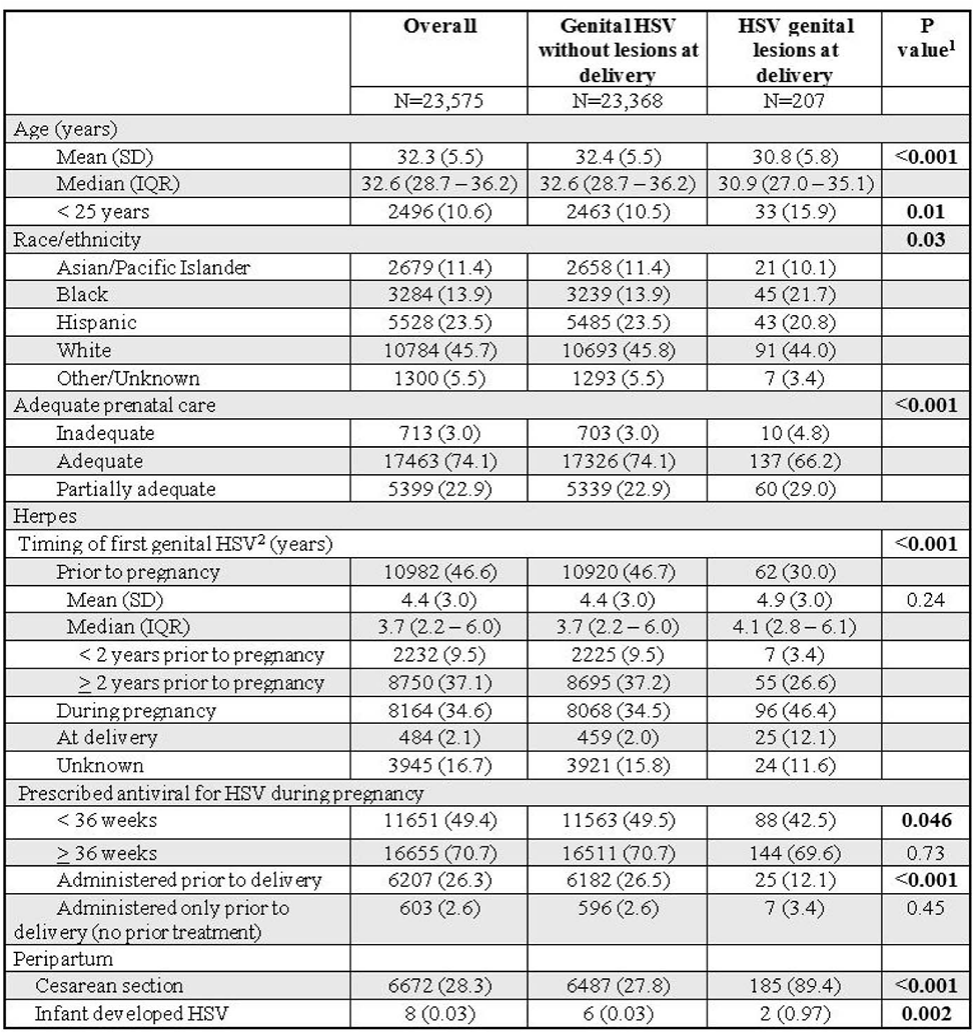
Pregnant persons with history of genital herpes and / or genital lesions at delivery delivering an infant >= 36 weeks gestation 1Differences between groups tested using Chi-Square or Fisher’s exact tests for categorical variables and Student’s t-test for continuous variables 2Timing of first genital HSV taken according to patients’ problem list

**Table 2:**
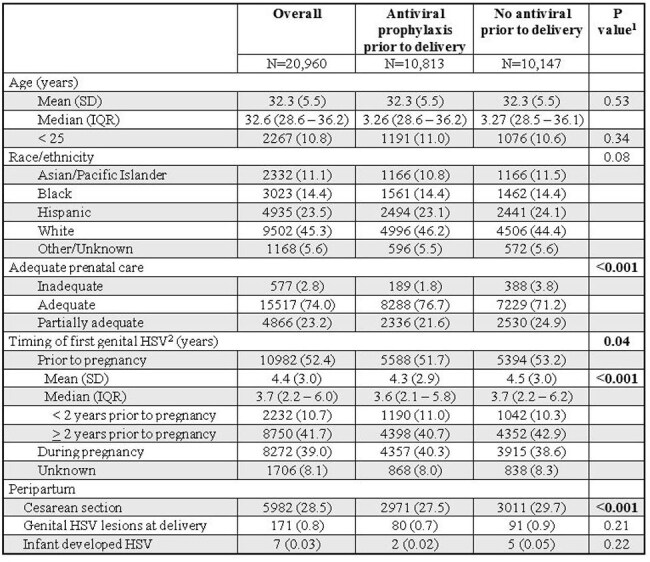
Outcomes of antiviral suppressive therapy >= 36 weeks gestation compared to no antiviral in pregnant persons with history of genital herpes 1Differences between groups tested using Chi-Square or Fisher’s exact tests for categorical variables and Student’s t-test for continuous variables 2Timing of first genital HSV taken according to patients’ problem list

**Methods:**

This is an observational, data-only retrospective study of all pregnant persons with an infant born from 10/01/2011 to 09/30/2023 who had a diagnosis prior to or during pregnancy of genital herpes and / or genital lesions at delivery. We extracted their demographics, timing of first genital HSV and proportion who received antenatal antiviral prophylaxis. We also described HSV infections in infants age 0-42 days born to these persons.

**Results:**

During our study period, there were a total of 480,991 pregnancies. Of those, 459,553 (95.54%) delivered 464,587 infants at or after 36 weeks gestation. In this cohort, 23,368 (5.1%) pregnant persons had a history of genital HSV without lesions at delivery and 207 (0.04%) had genital HSV lesions at delivery (Table 1). Pregnant persons with genital HSV lesions were younger, and more likely to be black race, have inadequate / partially adequate prenatal care, and diagnosed with HSV during pregnancy, and less likely to receive antenatal antiviral prophylaxis. Eight (0.03%) infants developed neonatal HSV, including 2 asymptomatic infants born to mothers with active genital HSV lesions screened for HSV per protocol.

Of the 20,960 pregnant persons with genital HSV diagnosed prior to delivery, 51.5% received antiviral prophylaxis at or after 36 weeks. Persons without antiviral therapy were more likely to have inadequate / partially adequate prenatal care, be diagnosed with genital HSV prior to pregnancy and have a cesarean section (Table 2). There was no difference in rates of neonatal HSV based on receipt of maternal antiviral.

**Conclusion:**

Maternal characteristics are associated with rates of HSV lesions at delivery, cesarean delivery, and neonatal HSV. Among these associations, receipt of antenatal antiviral prophylaxis may be modifiable through care delivery improvements.

**Disclosures:**

Tara L. Greenhow, MD, Moderna Therapeutics, Inc.: Grant/Research Support

